# Acute ulnar nerve compression associated with pisiform fracture – a case report and literature review

**DOI:** 10.1080/23320885.2018.1522959

**Published:** 2019-01-28

**Authors:** Min Kai Chang, Robert Tze Jin Yap

**Affiliations:** a Duke-NUS Medical School, Singapore, Singapore;; b Department of Hand Surgery, Singapore General Hospital, Singapore, Singapore

**Keywords:** Carpal, fracture, Guyon’s canal, pisiform, pisiformectomy, ulnar nerve

## Abstract

We evaluated a case of pisiform fracture with ulnar nerve compression managed with pisiformectomy. At 11 months’ follow-up, the patient regained range of motion of the wrist and grip strength with no subjective loss of function and normal nerve conduction study. We compared other treatment modalities and reviewed their outcomes.

## Introduction

Pisiform fractures are uncommon, with an average incidence that ranges from 0.2% to 2% of carpal fractures [5]. Due to pisiform’s proximity to the ulnar nerve in the Guyon canal, ulnar nerve palsy may occur when the pisiform fractures as a result of direct compression by fracture fragments or haematoma formation. Despite that, case reports of closed pisiform fracture that resulted in lower ulnar nerve palsy were rarely reported in literature. Due to the rarity of such cases, the management and functional outcomes are not well established. Treatment options for pisiform fracture include conservative immobilization with casting, closed reduction, open reduction with internal fixation, and pisiform excision. Pisiform excision has been shown to improve pain [3], but there have been varied reports on the functional outcomes of the wrist and ulnar nerve neuropraxia. We report a case of an acute ulnar nerve palsy as a consequence of an isolated closed pisiform fracture that was treated with pisiform excision. We also reviewed the literature on ulnar nerve recovery after the various forms of treatment options.

## Methods

### Case Report

**Figure 1.**
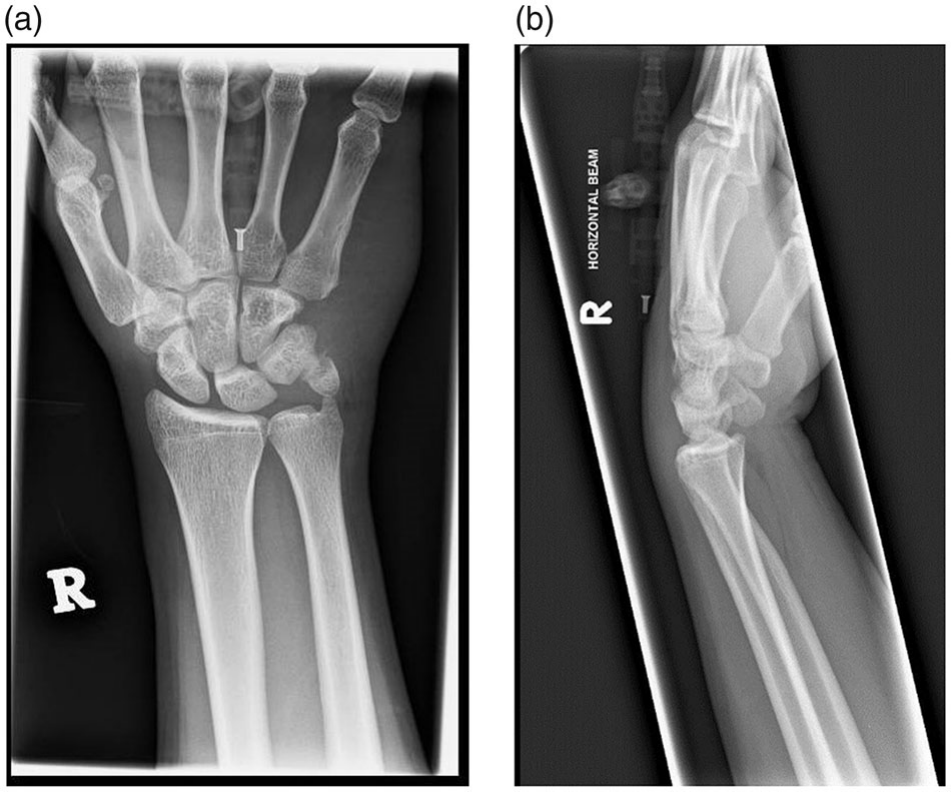
(a) X-ray (anteroposterior view) of the right hand, showing the displaced pisiform fracture. (b) X-ray (lateral view) of the right hand, showing the displaced pisiform fracture.

The patient is a 24-year-old right hand dominant rope access technician who was involved in a road traffic accident. His motorcycle had collided with the back of a truck, resulting in him being thrown forward and using his hands to shield from impact. His injuries included diastasis of pubic symphysis and undisplaced fracture of head of right radius, for which conservative measures were sufficient. He also had a left closed undisplaced scaphoid fracture and a right pisiform fracture (Figure 1(a,b)). On initial assessment, the pain in the both hands were masked by the other injuries. However, on detailed examination, he was noted to have paraesthesia in the ring and little fingers as well as weakness of the intrinsic muscles of the right hand. Froment’s and Wartenburg’s signs were positive.

**Figure 2.**
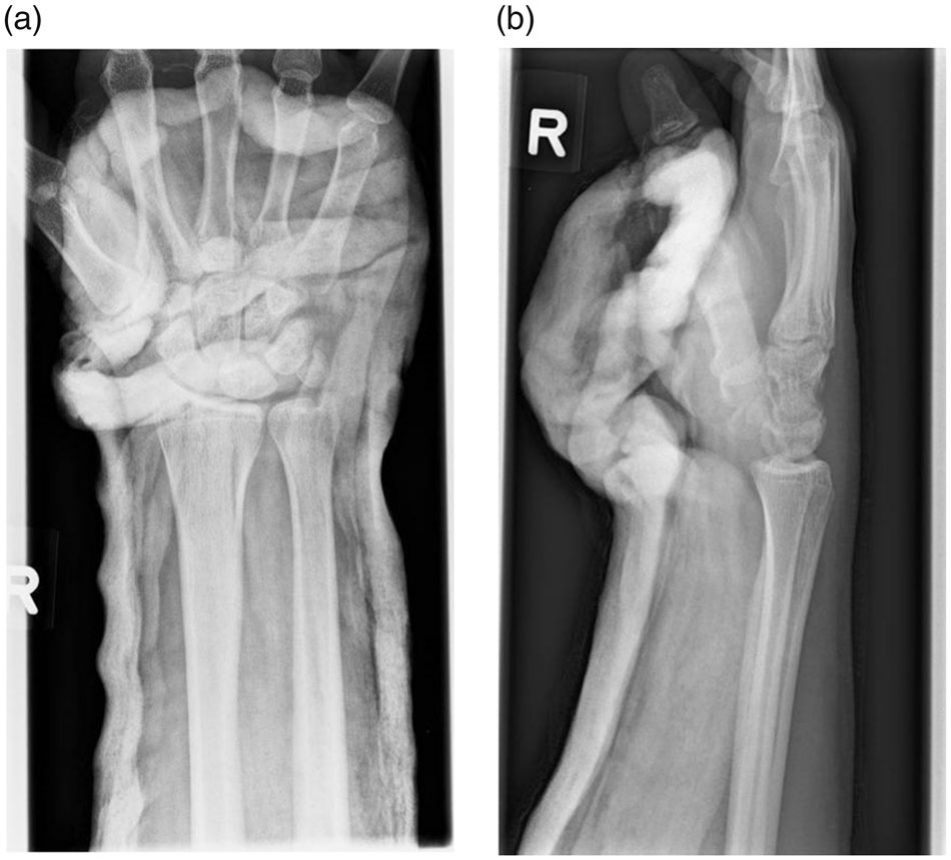
(a) X-ray (anteroposterior view) of the right hand after an unsuccessful manual closed reduction. (b) X-ray (lateral view) of the right hand after an unsuccessful manual closed reduction.

Manual closed reduction of the pisiform under anaesthesia was attempted at the bedside the next day. Post-reduction radiographs did not show satisfactory alignment (Figure 2(a,b)). In view of the acute onset of ulnar nerve deficit the patient was counselled for and underwent surgical exploration and ulnar nerve decompression at Guyon canal of the right hand. He also had the left scaphoid fixed as well in the same sitting.

**Figure 3.**
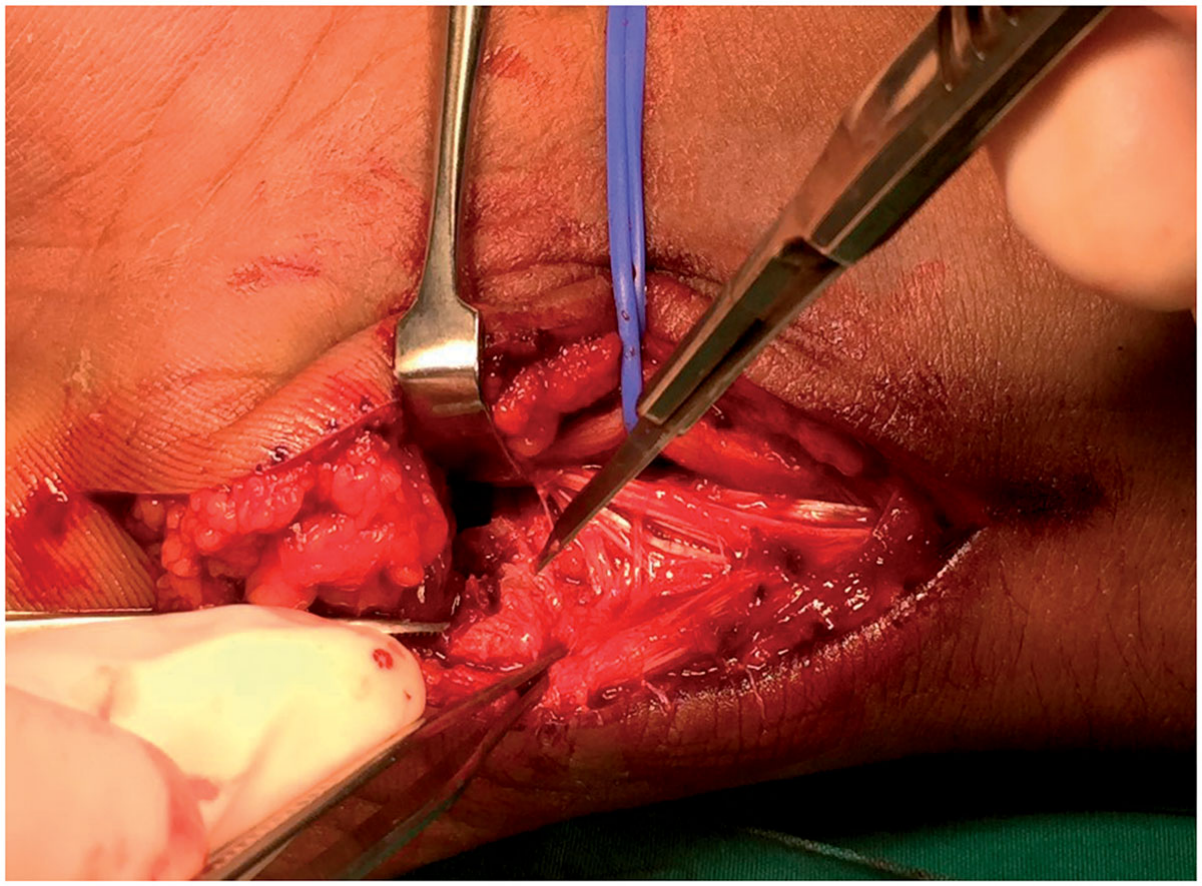
Decompression of the ulnar nerve. The proximally displaced fragment of the pisiform is indicated by the scalpel.

**Figure 4.**
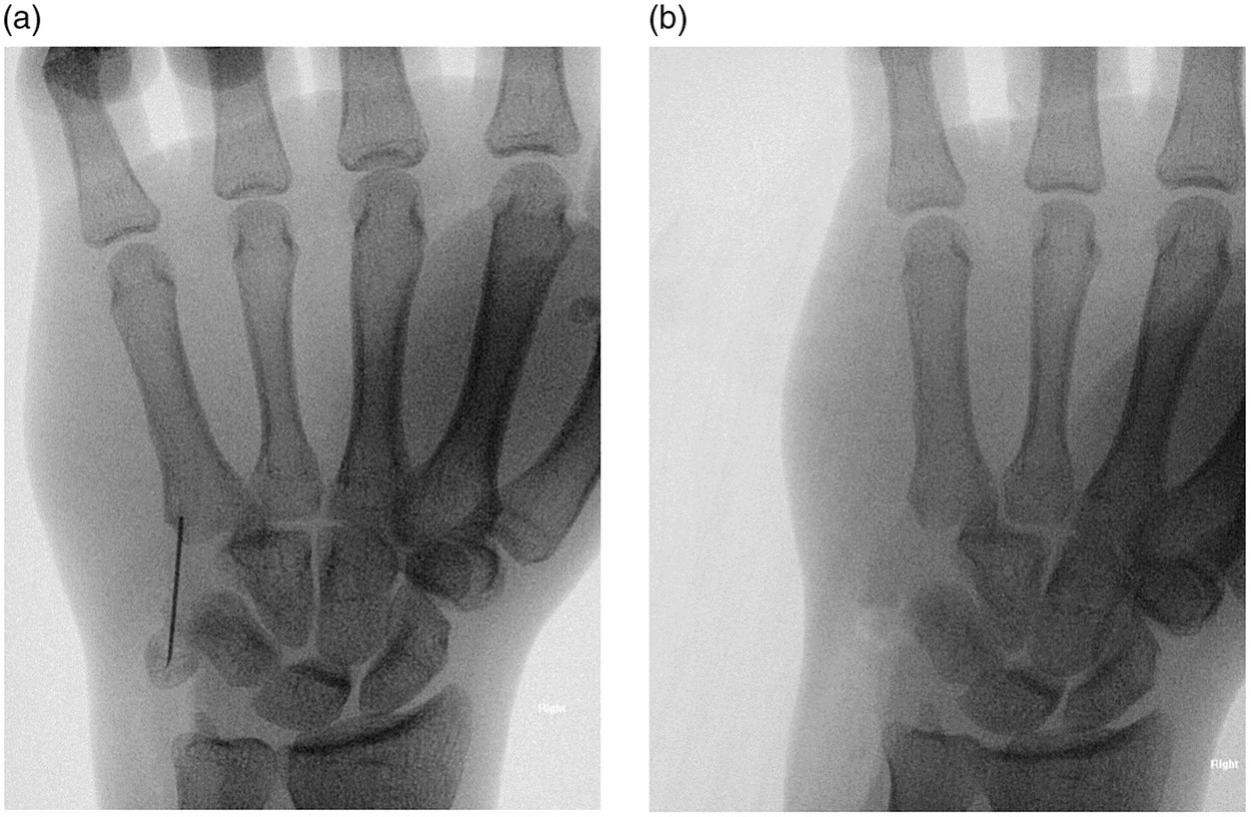
(a) X-ray (anteroposterior view) of the right hand before complete excision and removal of the comminuted pisiform. (b) X-ray (lateral view) of the right hand after complete excision and removal of the comminuted pisiform.

Intraoperatively, the ulnar nerve was found to be in continuity and contused over the fracture site at zone 1, proximal to the bifurcation of the ulnar nerve (Figure 3). The ulnar artery was intact and protected. The pisiform fracture was comminuted with the distal fragment attached to the pisohamate and pisometacarpal ligament. The distal and proximal fragments of the pisiform were removed, and the Guyon canal was decompressed (Figure 4(a,b)).

### Literature Search

A thorough literature search was performed to look for relevant case reports on pisiform fractures and ulnar nerve compression. Searches conducted focused on the term ‘pisiform fracture’. Searches were conducted using PubMed service available on the National Centre for Biotechnology Information (NCBI). Case reports of pisiform fracture and ulnar neurological deficit with management and outcomes were identified after reading through the whole articles.

## Results

Postoperatively, he had complete resolution of the ring and little finger numbness as well as restoration of the strength of the intrinsics. The patient was followed up till 11 months’ after the surgery. Notably, Froment’s and Wartenburg’s signs were both negative. Tinel’s test was negative at the injury site. The Jamar grip strength was 36 kgf for his right hand. He could achieve up to 40^°^ of dorsiflexion, 40^°^ of palmar flexion, 90^°^ of supination, 80^°^ of pronation, 10^°^ of ulnar deviation, and 15^°^ of radial deviation of the right wrist. The static two-point discrimination test for the ulnar part of the ring finger and both radial and ulnar part of the little finger was less than 5mm. Disabilities of the Arm, Shoulder, and Hand (DASH) questionnaire was also administered to measure physical function. The score was 0 out of 100. Nerve conduction study showed no electrophysiological evidence of ulnar neuropathy with conduction velocity of 57.3 m/s for below elbow stimulation (58.0m/s for left hand). Patient refused electromyography study due to pain involved in the needle insertion.

Out of a total of 31 relevant case studies on pisiform fractures, only 4 case reports indicated ulnar neuropathy following acute pisiform fracture and included management plans with outcomes following treatment [1,6,7,9]. The management and outcomes were varied as seen in Table 1.

**Table 1:** Case reports of pisiform fracture with ulnar nerve compression and the related treatment and outcomes.

	Authors	Injury Mechanism	Neurological Examination	Diagnosis	Treatment	Outcomes
1	Howard F (1961)^3^	Fall on outstretched right hand	Sensory: numbness of ring and little fingers Motor: right grip 55% compared to left, weakness of first dorsal interosseous muscle, decreased digital spread of 2cm between little and ring fingers, weak little finger abduction	Pisiform fracture	No immobilisation; active motion begun immediately	6 months: positive Tinel sign, span of right hand still diminished, normal first interosseous muscle strength
2	Israeli A et al. (1982)^4^	Repetitive trauma from volleyball	Sensory: hypoesthesia on the ulnar aspect of the little finger Motor: intact	Pisiform fracture	3 weeks of plaster immobilization, 3 weeks of rest	6 weeks: complete neurological and functional recovery, mild effort induced pain in hypothenar area
3	Matsunaga D et al. (2002)^5^	Fall from 5m height and sustained multiple injuries Case 1: left hand injury Case 2: right hand injury	Case 1 Sensory: paraesthesia in little finger, ulnar side of ring finger, ulnar side of palm Motor: no weakness in intrinsic muscles, Grip strength 20kg (L), 38kg (R) Case 2 Sensory: paraesthesia in little finger, ulnar side of ring finger, ulnar side of palm Motor: severe muscle atrophy of interosseous muscles, claw finger deformity of right ring and little fingers, grip strength 31kg (L), 5kg (R), pinch strength 8kg (L), 2.8kg (R)	Case 1: Pisiform fracture non-union with compression of the distal Guyon's canal Case 2: Pisiform fracture with compression of the distal Guyon's canal	Pisiformectomy + external neurolysis	Case 1 3 months: decrease in numbness 21 months: no numbness, no sensory disturbance, NCS normal, grip strength 40kg (left), 40kg (right) Case 2 3 months: improvement of numbness 19 months: full motor recovery with slight numbness in ring and little fingers, range-of-motion of wrist normal without pain
4	Agathangelidis F et al. (2013)^6^	Fall on broken glass (left hand)	Sensory: numbness of little finger Motor: intact	Isolated displaced pisiform fracture and bruising of ulnar nerve	Internal fixation with 2.5 cortical screw, enforced by cerclage of the bone with No 5 Ethibond suture	14 weeks: complete fracture union 6 months: painless motion and acceptable degree of hand function

## Discussion

The pisiform is a sesamoid bone in the proximal carpal row, located on the volar and ulnar aspect of the wrist. It has been thought to have several functions. Firstly, it forms the ulnar border of the Guyon canal, where ulnar nerve and artery course. It has been suggested that due to this close relation with the ulnar nerve and artery, the pisiform protects the neurovascular structures of the Guyon canal [2]. Secondly, the pisiform lies within the fibres of the flexor carpi ulnaris. Although pisiform has no active role in the movement of the wrist joint, it increases the flexor force of flexor carpi ulnaris by extending the lever arm of the tendon away from the centre of rotation of the wrist. Thirdly, the pisiform is part of the pisiform ligament complex, a group of ligaments attached to the pisiform. It contributes to the stability of the wrist ulnar column and it could play a role in the biomechanics of the ulnar wrist [2].

These postulated functions of the wrist suggest that the surgical removal of the pisiform, even with minimal disruption to its soft tissue attachments, could affect the functions of the wrist. Beckers and Koebke [2] highlighted that the stability of the pisotriquetral joint is controlled by nine important structures that may be damaged by pisiform excision, and have thus cautioned against pisiform excision. Cadaveric studies performed by O’Keefe et al. [10] revealed that in simulated high-demand wrist activities, greater flexor carpi ulnaris forces are required after pisiform excision, hence it may be a concern in patients who engage in high-demand activities.

On the other hand, there are also significant number of reports that found that there is no functional loss of the wrist after pisiform excision. Campion et al. [3] showed that pisiform excision has no long term adverse effects on the wrist range of motion and strength if the pisiform ligaments are preserved. Van Ejizeren et al. [13] reported that there is no significant difference in the objective assessment of the operated and nonsurgical wrists, which included grip and pinch strength, flexion, ulnar and radial deviation. Majority of the patients returned to their previous levels of employment and other activities or hobbies. In a review publication, Suh et al. [12] reported that pisiform excision can provide excellent results with no loss of range of motion and reliable relief of pain for displaced pisiform fractures. Another literature review by O’Shea et al. [11] revealed that pisiform excision is a safe procedure for displaced fracture. Evidence of pisiform excision affecting the ulnar nerve is scarce. Van Ejizeren et al. [13] reported that 8 out of 10 patients with ulnar nerve neuropraxia continued to have ulnar symptoms in the early post-operative phase of 1 to 6 weeks, but subsequently the neuropraxia resolved, suggesting that neuropraxia is transient with careful protection. Carroll et al. [4] reported that all 22 patients with mild ulnar paraesthesia and/or hypoesthesia had full sensory recovery after undergoing neurolysis and pisiform excision. 5 out of 6 patients with intrinsic motor weakness had full motor recovery and 1 patient had partial motor recovery due to chronic intrinsic muscular atrophy. However, Campion et al. [3] showed that 2 out of 3 patients who underwent ulna nerve decompression with pisiform excision continued to suffer from ulnar symptoms after 16 and 19 months postoperatively. They cautioned that preoperative ulnar nerve problems may not be relieved if severe compression and nerve damage are present. To our knowledge, no study has been done to investigate the duration and degree of ulnar nerve injury in the pre-operative state and the outcomes after pisiform excision with ulnar nerve decompression.

Howard [6] advocated that immobilization may not be necessary initially, but if prompt recovery of nerve function is not apparent, the ulnar nerve should be explored in six to eight weeks. He presented a case of displaced pisiform fracture with immediate active mobilization; at six months’ follow-up, there is recovery of the strength of the first interosseous muscle, but Tinel’s sign is still positive and span of right hand continues to be diminished. Israeli et al. [7], on the other hand, presented one case of pisiform fracture with ulnar nerve compression that was immobilized with plaster for 3 weeks, followed by 3 weeks of rest. At three weeks’ follow up, there was complete neurological and functional recovery with mild effort induced pain in the hypothenar area. Matsunaga et al. [9] explored the distal ulnar tunnel with excision of entire pisiform bone for two cases: one non-union of pisiform fracture and one comminuted fracture; at 19 and 21 months’ follow-up respectively, there was slight numbness in the ring and little fingers for one of the cases, but no weakness of the intrinsic muscles in both cases. Agathangelidis et al. [1] presented a case of open pisiform fracture that was managed by internal fixation with cerclage. At 14 weeks, there was complete fracture union, and at 6 months’ follow-up, there was painless motion with ‘acceptable degree of hand function’, but no indication in the recovery of the ulnar nerve. These reports demonstrated the range of therapeutic options with varied results.

We present a case of ulnar nerve neuropraxia secondary to pisiform fracture that was first managed by closed manual reduction under anaesthesia, then pisiform excision with ulnar nerve decompression. Notably, the ulnar nerve symptoms were not evident in the initial assessment, indicating an acute progressive neurological deficit or that other concomitant injuries may have masked the symptoms. To avoid chronic complications of ulnar nerve compression and to appropriately manage the patient, it is prudent to adequately evaluate the ulnar neurovascular function in the initial screening. This may involve examining under adequate anaesthesia and appropriate imaging modalities. If routine radiographs in anteroposterior, lateral, pronated oblique views fail to demonstrate pisiform fracture, and clinical examination is ambiguous for ulnar nerve injury, magnetic resonance imaging can be considered.

For our case, unfortunately, objective tests could not be compared with the left hand because of the recovering scaphoid fracture. These tests performed within 11 months’ follow-up showed that the grip strength of the right hand was 36 kgf, which is within one standard deviation of the grip strength of normal right-handed 24-year-old adult [8]. The range of motion of the right wrist fell within the functional range of motion. Neurological examination showed that neuropraxia is temporary, with the patient regaining full sensory and motor function. DASH questionnaire of the physical function of the patient revealed that there was no subjective disability of the injured hand.

## Conclusions

There are varied treatment options for acute ulnar nerve compression following pisiform fracture with different outcomes. Our case study showed that pisiformectomy after pisiform fracture with ulnar nerve compression produced good objective and subjective outcomes. In addition to the favourable outcomes, we believe that pisiform excision with ulnar nerve decompression is preferred over immobilization or the fixation of the pisiform, because adequate release of the ulnar nerve can be directly visualised. As reviewed above, most authors have reported that neuropraxia is transient when pisiform excision with ulnar nerve decompression is performed.
